# Supplementary material for: The Role of Chromatin Accessibility in cis-Regulatory Evolution
Source: Genome Biol Evol. 2019 May 22;11(7):1813–28. doi: 10.1093/gbe/evz103 (PMC6601868; doi:10.1093/gbe/evz103)
Supplement: Supplementary_Material_evz103 [file supplementary_material_evz103.zip › Attached file_ Supporting-information-legends.docx]

**SUPPORTING INFORMATION LEGENDS**

**Table S1. Relationship of accessibility with interspecies ChIP changes.** Pairwise Pearson correlation coefficients (CC) of ΔChIP of TF1 and TF2 at the same time point ‘x’ are shown, and partial correlation coefficients after excluding the effect of accessibility. Bold fonts indicate cases where the difference between CC and partial CC is at least 0.04.

**Table S2. Classifier predictions of enhancer activity agree with results of transgenic reporter assays reported in previous studies.** Predicted activities are compared with spatio-temporal expression in three classes: mesoderm (Meso), visceral muscle (VM), and somatic muscle (SM). An enhancer activity class is assigned if the respective classifier prediction value is greater than 0.9. Enhancers in D. melanogaster (Dmel) and D. virilis (Dvir) were previously tested for in-vivo activity in D. melanogaster embryos.

**TableS3. Enhancer activity predictions from Support Vector Machine in Zinzen et al. for the 233 experimentally characterized enhancers.** Balanced accuracy is shown for SVM models built for each activity class: mesoderm (‘Meso’), visceral muscle (‘VM’), and somatic muscle (‘SM’). According to Zinzen et al., an enhancer is classified to an activity class if the SVM specificity is greater than 95%. The numbers of correctly and incorrectly classified enhancers for each model are listed. TN: true negative, FN: false negative, TP: true positive, FP: false positive.

**Figure S1. Position weight matrices (PWMs) are shown in the form of sequence logos, source: [Khoueiry et al., eLife, 2017].**

**Figure S2. Scatter plots of D. melanogaster ChIP scores versus D. virilis ChIP scores for each TF:TP combination.** Points represent orthologous enhancers that are accessible in at least one species. Colors indicate change of accessibility score.

**Figure S3. AUROC measure of classification accuracy.** Predicted ΔChIP based on ΔAcc (pΔChIP(ΔAcc)) is used to classify enhancer pairs with the greatest increase in TF binding versus those with the greatest decrease in binding.

**Figure S4. STAP models accurately fit TF occupancy (ChIP) data in single species, either D. melanogaster (x-axis) or D. virilis (y-axis).** For each TF-time point condition, average Pearson correlation coefficient from 5-fold cross-validation is shown.

**Figure S5. Performance of accessibility-based predictors. (A)** Models using accessibility data from ChIP-matched stage perform nearly as well as models aggregating accessibility data from all available stages. We recognized that using data from multiple time points may inflate the strength of relationship between accessibility and binding changes, leading to unfair comparisons with motif-based predictions of ΔChIP. We therefore compared accessibility-based SVR models that use all three timepoints with models using only the time point matching the ChIP data. We found that for six of nine TF-TP conditions the two models yield equal correlations, while for three conditions the correlation is slightly better when utilizing multiple time points. (B) Performance of accessibility-based predictors is better at early time points. (Adopted from Figure 3B in main text, sorted by time points.)

**Figure S6. AUROC measure of classification accuracy.** Predicted ΔChIP based on motif changes and accessibility changes (pΔChIP(ΔSTAP+ΔAcc)) is used to classify enhancer pairs with the greatest increase in TF binding versus those with the greatest decrease in binding.

**Figure S7. ROC plots for delineating how well** $\hat{\boldsymbol{\Delta A}_{\mathbf{C}}}$ **values can classify high versus low ΔAC ­ enhancer pairs in each activity class.** $\hat{{\Delta A}_{C}}$ values are based on D. virilis ChIP score profiles imputed from D. melanogaster scores and predictions of binding change.

**Figure S8. Normalized ChIP scores in D. melanogaster and D. virilis show similar distributions for all 14 TF-time point conditions.**

**Figure S9. Change in enhancer activities.** Relationship between change of TF binding and model-based change of enhancer activity, examined through 223 experimentally characterized enhancers. For each spatiotemporal expression domain, D. melanogaster enhancers with experimentally validated activity in that domain are considered, along with their D. virilis orthologs. Enhancer pairs are divided into “High” and “Low” TF binding change, based on sum of ΔChIP scores for all TFs. Change in predicted enhancer activity (ΔA_C_ , see text) is then compared between these two classes.
