# Supplementary material for: The Role of Chromatin Accessibility in cis-Regulatory Evolution
Source: Genome Biol Evol. 2019 May 22;11(7):1813–28. doi: 10.1093/gbe/evz103 (PMC6601868; doi:10.1093/gbe/evz103)
Supplement: Supplementary_Material_evz103 [file supplementary_material_evz103.zip › DmelDvir_Supplement.docx]

**Table S1. Relationship of accessibility with interspecies ChIP changes.** Pairwise Pearson correlation coefficients (CC) of ΔChIP of TF1 and TF2 at the same time point ‘x’ are shown, and partial correlation coefficients after excluding the effect of accessibility. Bold fonts indicate cases where the difference between CC and partial CC is at least 0.04.

| **ΔTF1_TPx** | **ΔTF2_TPx** | **CC** | **Partial CC** |
| --- | --- | --- | --- |
| ΔMef2_TP2 | ΔTin_TP2 | **0.45** | 0.37 |
| ΔBin_TP3 | ΔMef2_TP3 | **0.32** | 0.25 |
| ΔTin_TP3 | ΔTwi_TP3 | **0.39** | 0.32 |
| ΔBap_TP3 | ΔMef2_TP3 | **0.38** | 0.32 |
| ΔMef2_TP3 | ΔTwi_TP3 | **0.34** | 0.29 |
| ΔTin_TP2 | ΔTwi_TP2 | **0.50** | 0.45 |
| ΔBap_TP3 | ΔTin_TP3 | **0.46** | 0.42 |
| ΔBap_TP3 | ΔBin_TP3 | **0.42** | 0.38 |
| ΔBap_TP3 | ΔTwi_TP3 | **0.41** | 0.37 |
| ΔBin_TP3 | ΔTwi_TP3 | **0.30** | 0.26 |
| ΔMef2_TP3 | ΔTin_TP3 | 0.42 | 0.38 |
| ΔBin_TP3 | ΔTin_TP3 | 0.37 | 0.34 |
| ΔBin_TP4 | ΔMef2_TP4 | 0.24 | 0.23 |
| ΔBin_TP5 | ΔMef2_TP5 | 0.19 | 0.19 |
| ΔMef2_TP2 | ΔTwi_TP2 | 0.30 | 0.31 |
| ΔMef2_TP1 | ΔTwi_TP1 | 0.21 | **0.27** |

**Table S2. Classifier predictions of enhancer activity agree with results of transgenic reporter assays reported in previous studies.** Predicted activities are compared with spatio-temporal expression in three classes: mesoderm (Meso), visceral muscle (VM), and somatic muscle (SM). An enhancer activity class is assigned if the respective classifier prediction value is greater than 0.9. Enhancers in *D. melanogaster* (Dmel) and *D. virilis* (Dvir) were previously tested for *in-vivo* activity in *D. melanogaster* embryos.

| **Species_CRM_ID** | **Experimental essay** | **Predicted activity** | **Prediction**  **correct/failed** |
| --- | --- | --- | --- |
| Dmel_CRM_404 | Meso | Meso | correct |
| Dmel_CRM_633 | Meso | - | failed |
| Dmel_CRM_2000 | Meso | Meso | correct |
| Dmel_CRM_2045 | Meso | Meso | correct |
| Dmel_CRM_3407 | Meso | Meso | correct |
| Dmel_CRM_4682 | Meso | - | failed |
| Dmel_CRM_5278 | Meso | Meso | correct |
| Dmel_CRM_6053 | Meso | Meso | correct |
| Dmel_CRM_6176 | Meso | SM | failed |
| Dmel_CRM_388 | Meso weak | - | failed |
| Dmel_CRM_965 | SM | SM | correct |
| Dmel_CRM_1195 | SM | - | failed |
| Dmel_CRM_3215 | SM | SM | correct |
| Dmel_CRM_4575 | SM | SM | correct |
| Dmel_CRM_4725 | SM | SM | correct |
| Dmel_CRM_3027 | SM, VM weak | SM | partially correct |
| Dmel_CRM_160 | VM | VM | correct |
| Dmel_CRM_1560 | VM | - | failed |
| Dmel_CRM_2347 | VM | VM | correct |
| Dmel_CRM_2819 | VM | VM | correct |
| Dmel_CRM_3418 | VM | VM | correct |
| Dmel_CRM_4726 | VM | VM | correct |
| Dmel_CRM_4906 | VM | VM | correct |
| Dmel_CRM_5570 | VM | VM | correct |
| Dmel_CRM_6028 | VM | VM | correct |
| Dmel_CRM_6087 | VM | VM | correct |
| Dvir_CRM_4357 | Meso | - | failed |
| Dvir_CRM_14133 | Meso | - | failed |
| Dvir_CRM_12291 | Meso, SM, VM | SM | partially correct |
| Dvir_CRM_10323 | SM | SM | correct |
| Dvir_CRM_12156 | SM | SM | correct |
| Dvir_CRM_11115 | SM, VM | SM | partially correct |
| Dvir_CRM_459 | VM | VM | correct |
| Dvir_CRM_2469 | VM | SM | failed |
| Dvir_CRM_13100 | VM | VM | correct |

**Table S3. Enhancer activity predictions from Support Vector Machine in Zinzen et al. for the 233 experimentally characterized enhancers.** Balanced accuracy is shown for SVM models built for each activity class: mesoderm (‘Meso’), visceral muscle (‘VM’), and somatic muscle (‘SM’). According to Zinzen et al., an enhancer is classified to an activity class if the SVM specificity is greater than 95%. The numbers of correctly and incorrectly classified enhancers for each model are listed. TN: true negative, FN: false negative, TP: true positive, FP: false positive.

|  | **Meso** | **VM** | **SM** |
| --- | --- | --- | --- |
| **TN** | 130 | 157 | 163 |
| **FN** | 93 | 40 | 48 |
| **TP** | 9 | 25 | 18 |
| **FP** | 1 | 11 | 4 |
| **Balanced Accuracy** | 0.54 | 0.66 | 0.62 |


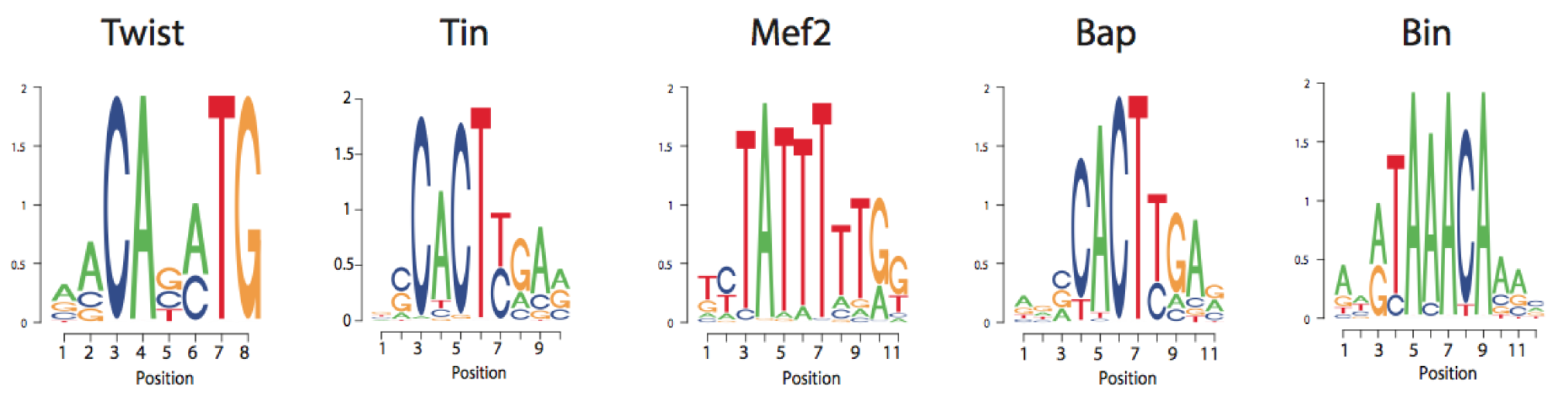


**Figure S1. Position weight matrices (PWMs) are shown in the form of sequence logos**, source: [Khoueiry et al., eLife, 2017].


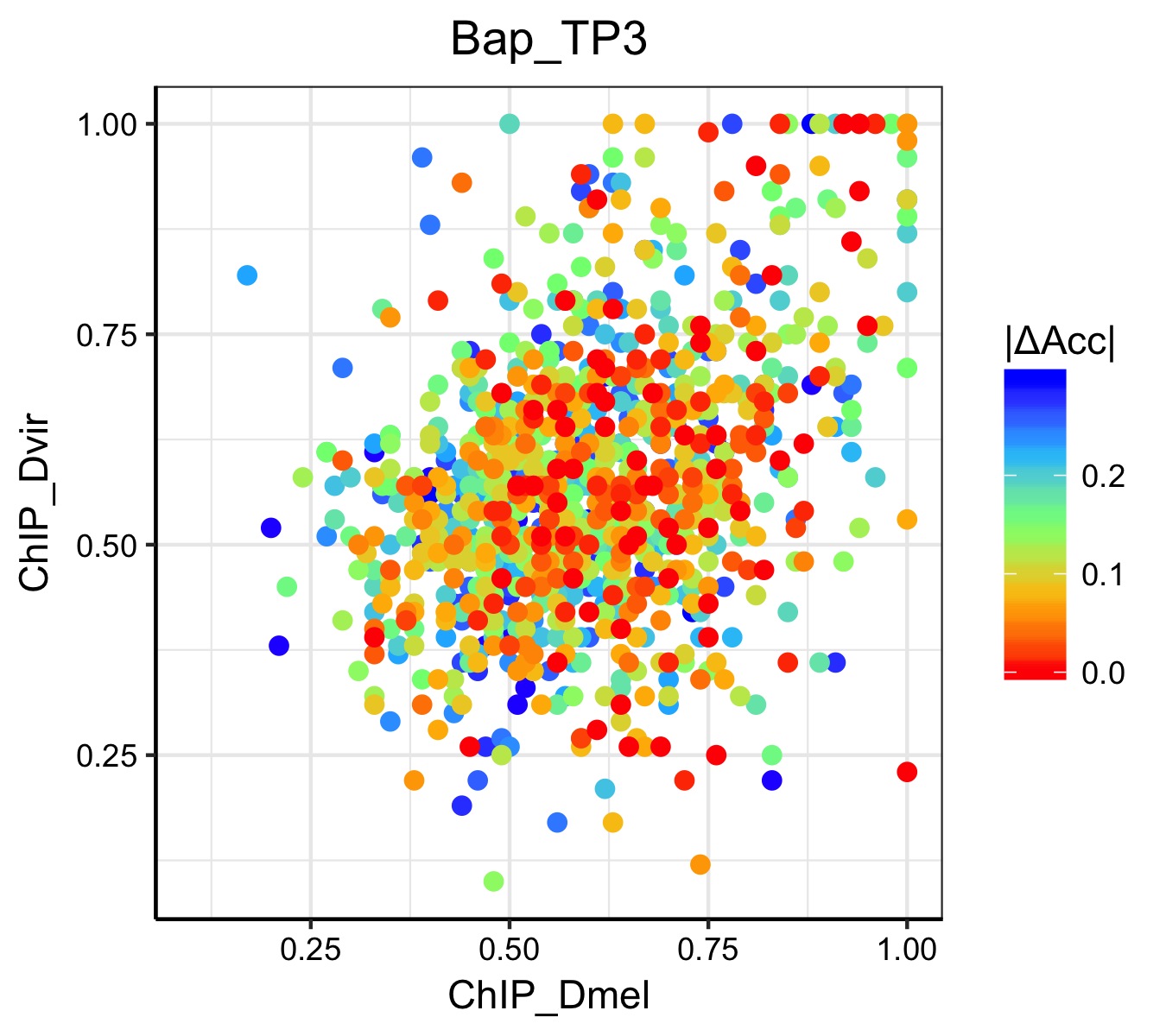

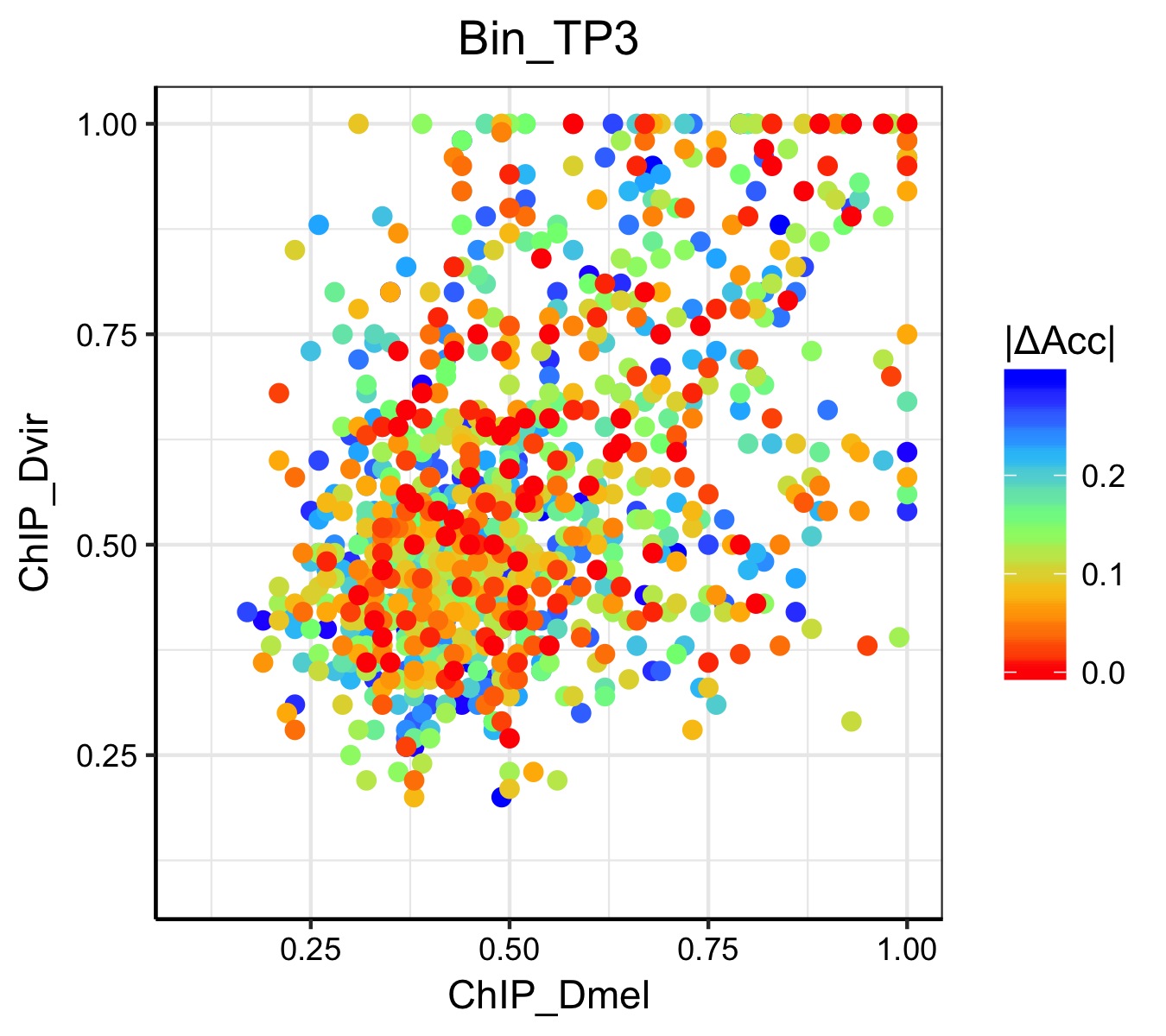

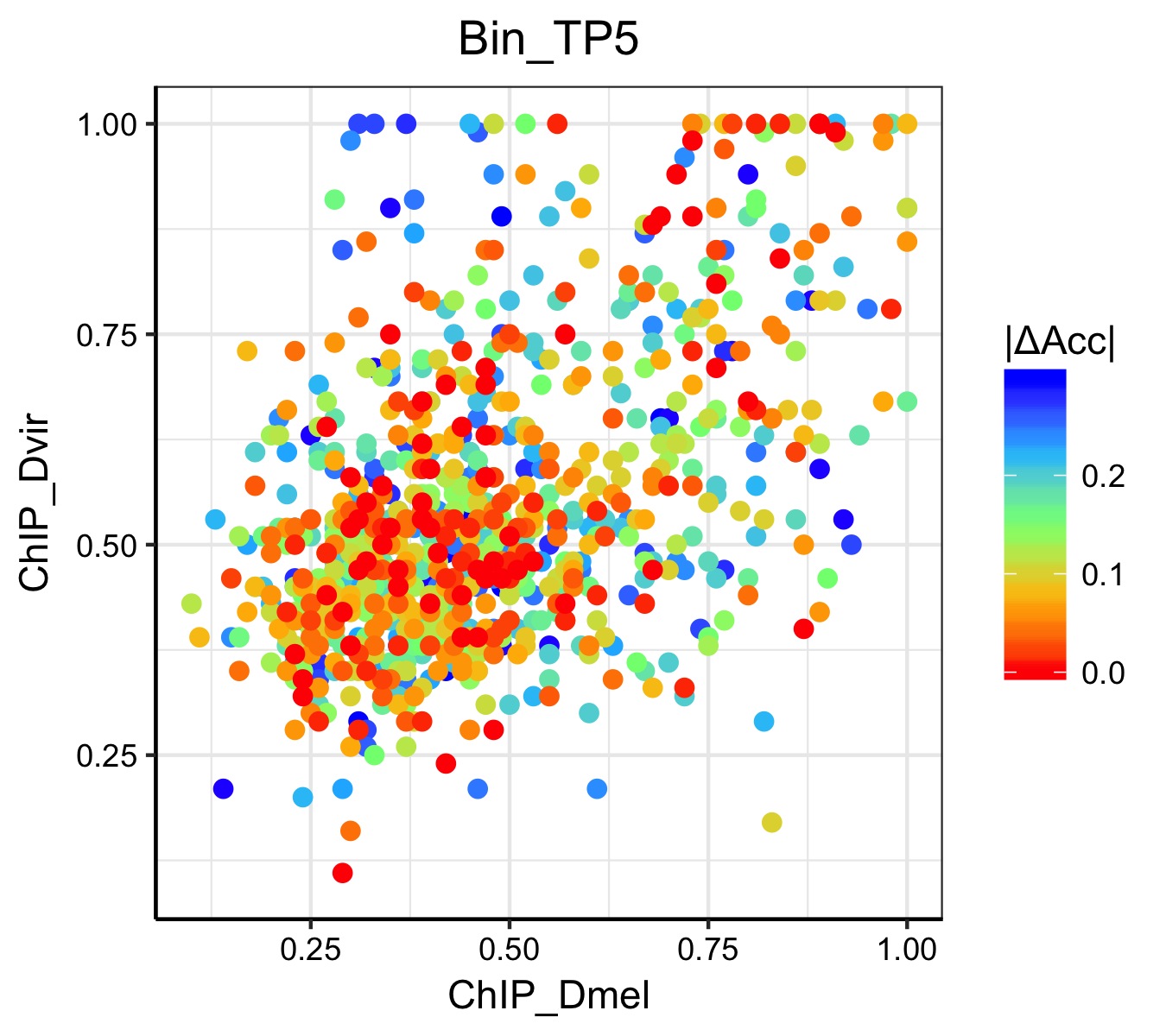

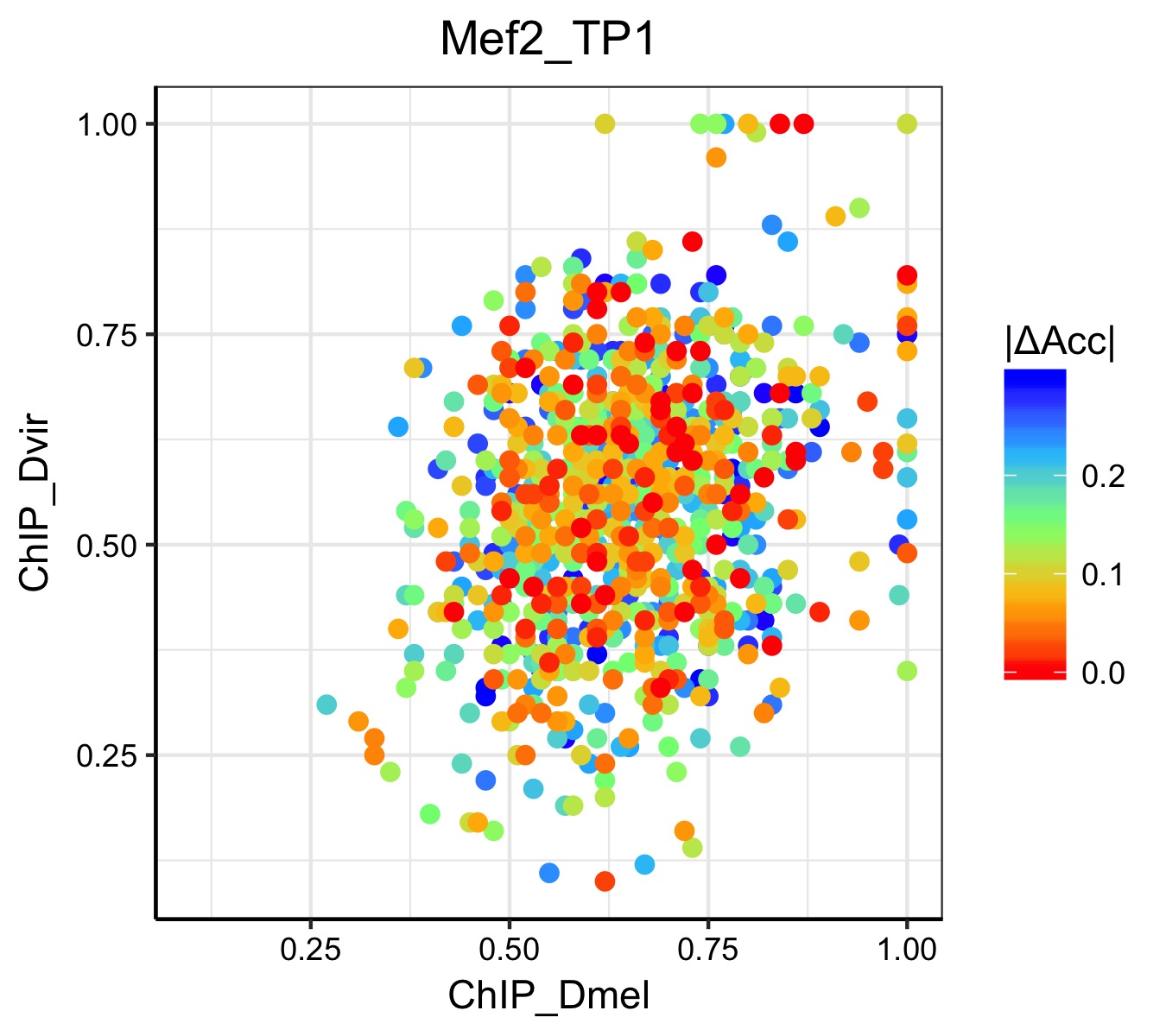

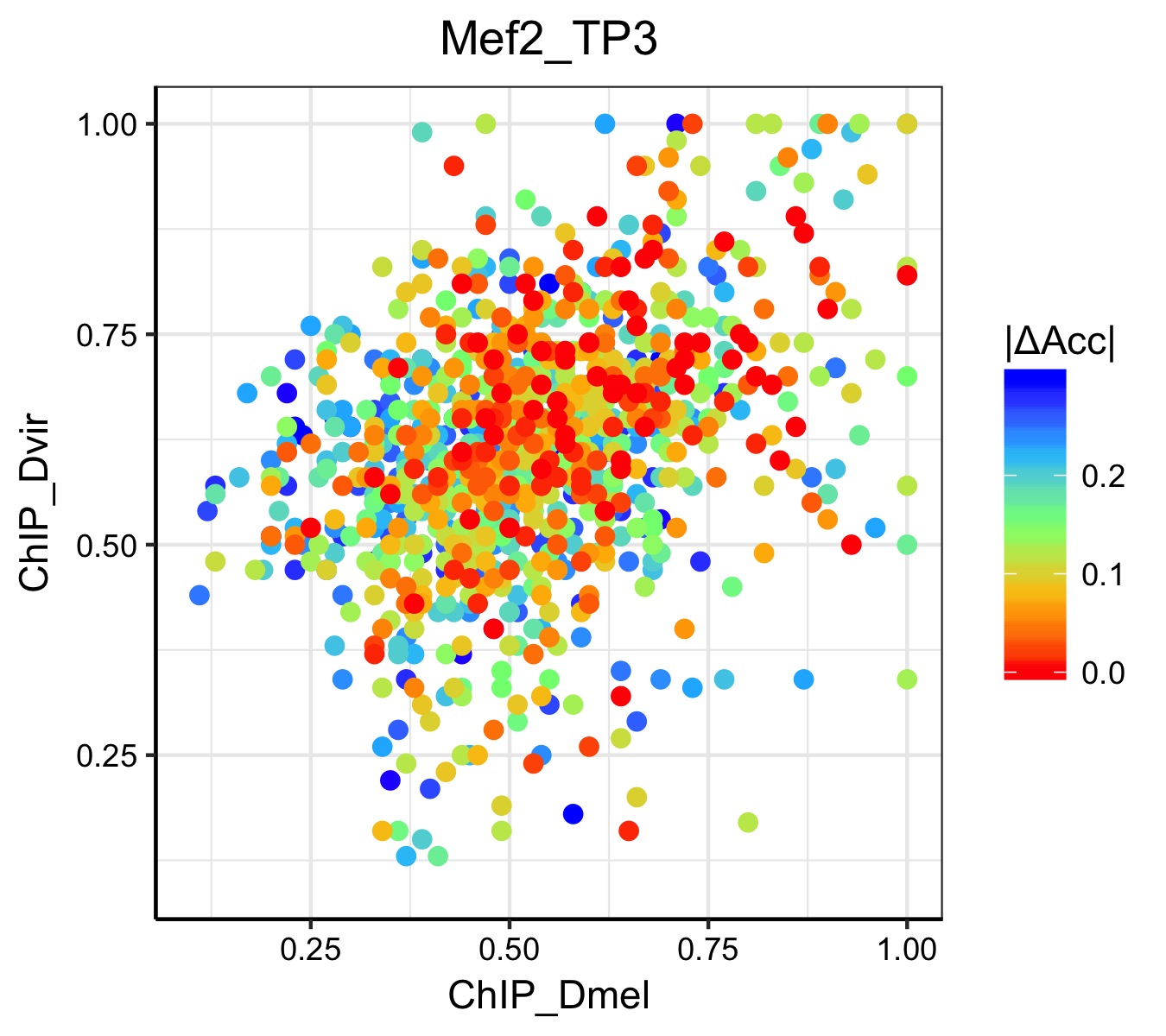

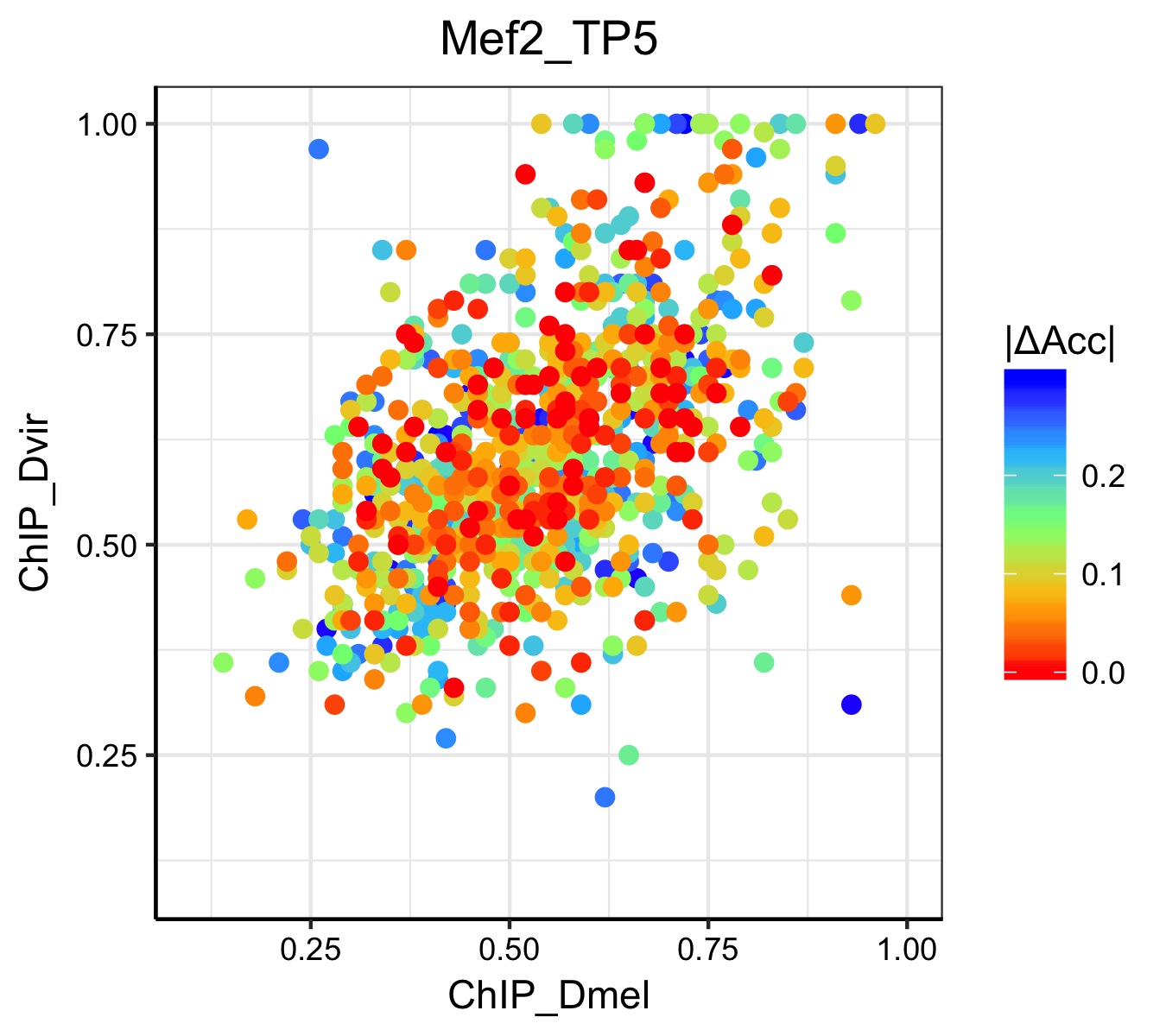

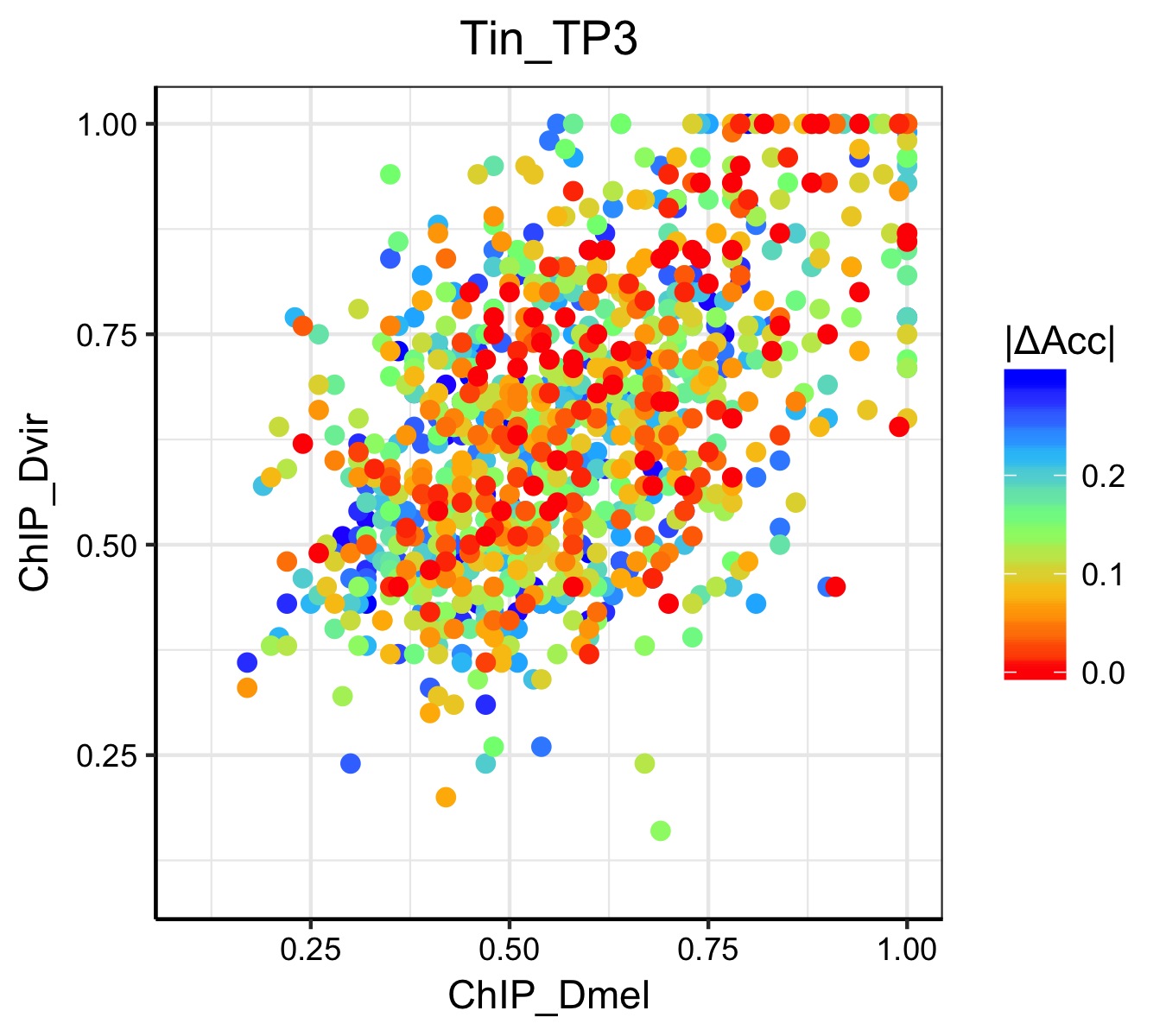

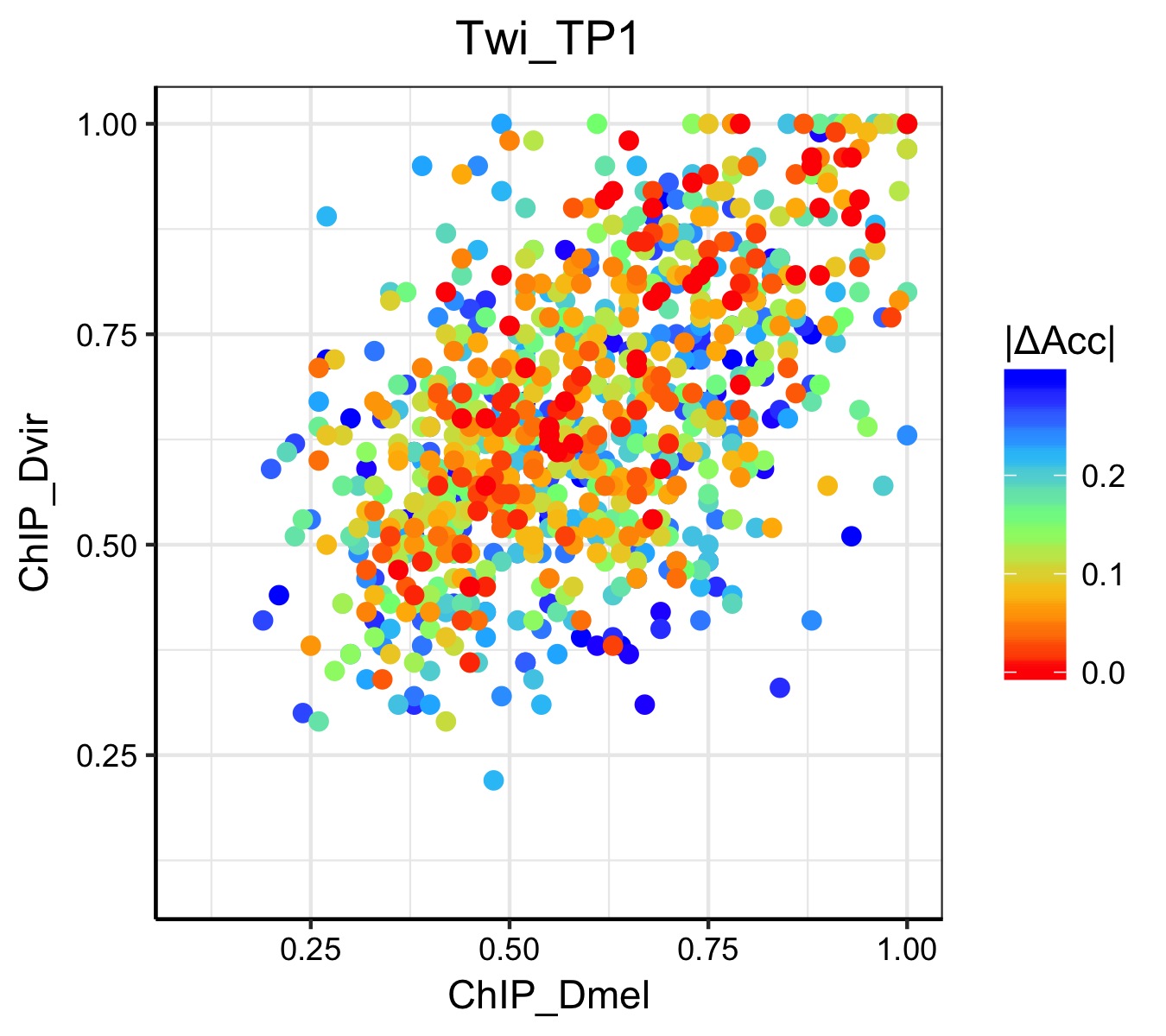

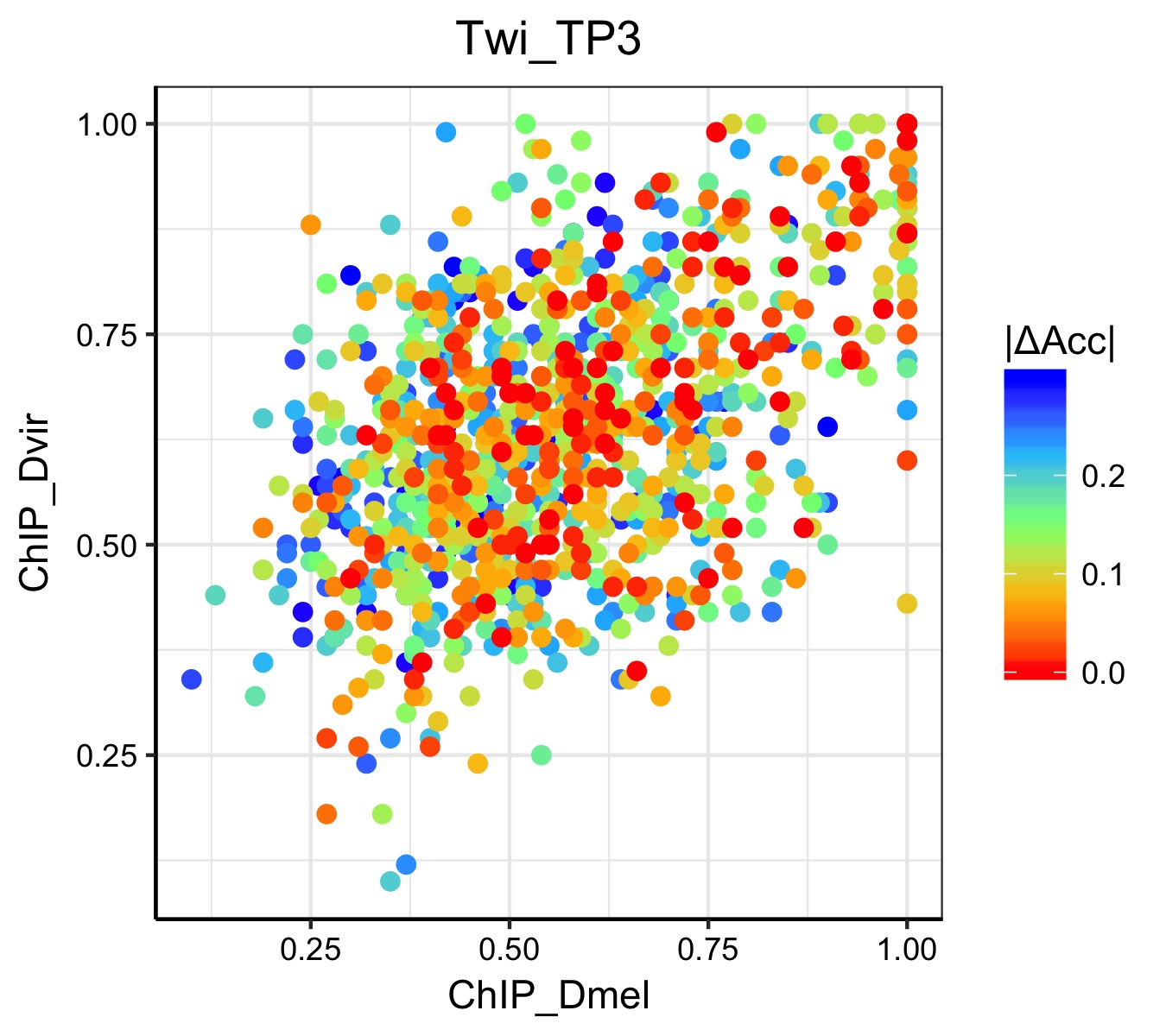


**Figure S2**. **Scatter plots of *D. melanogaster* ChIP scores versus *D. virilis* ChIP scores for each TF:TP combination.** Points represent orthologous enhancers that are accessible in at least one species. Colors indicate change of accessibility score.


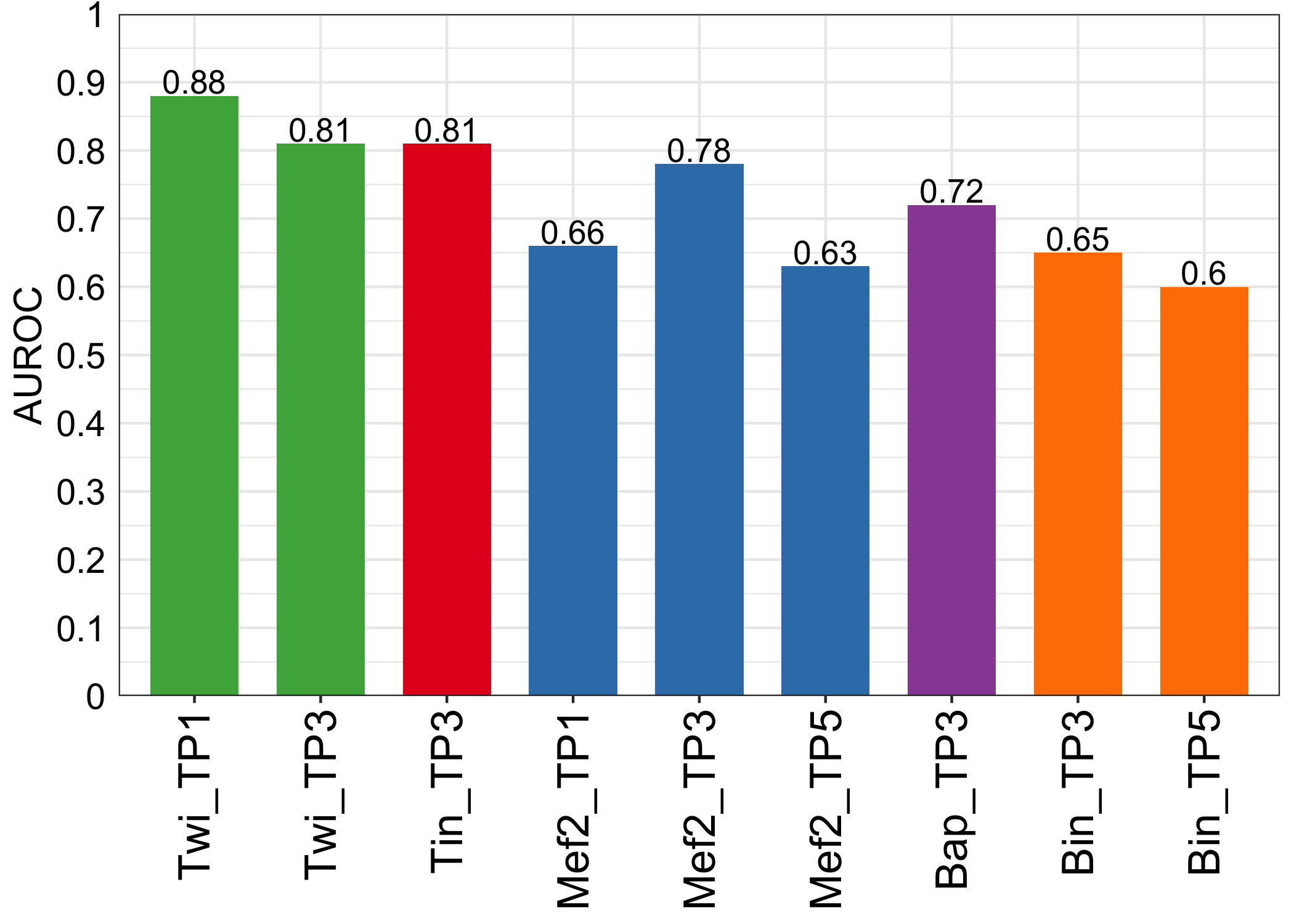


**Figure S3. AUROC measure of classification accuracy.** Predicted ΔChIP based on ΔAcc (pΔChIP(ΔAcc)) is used to classify enhancer pairs with the greatest increase in TF binding versus those with the greatest decrease in binding.


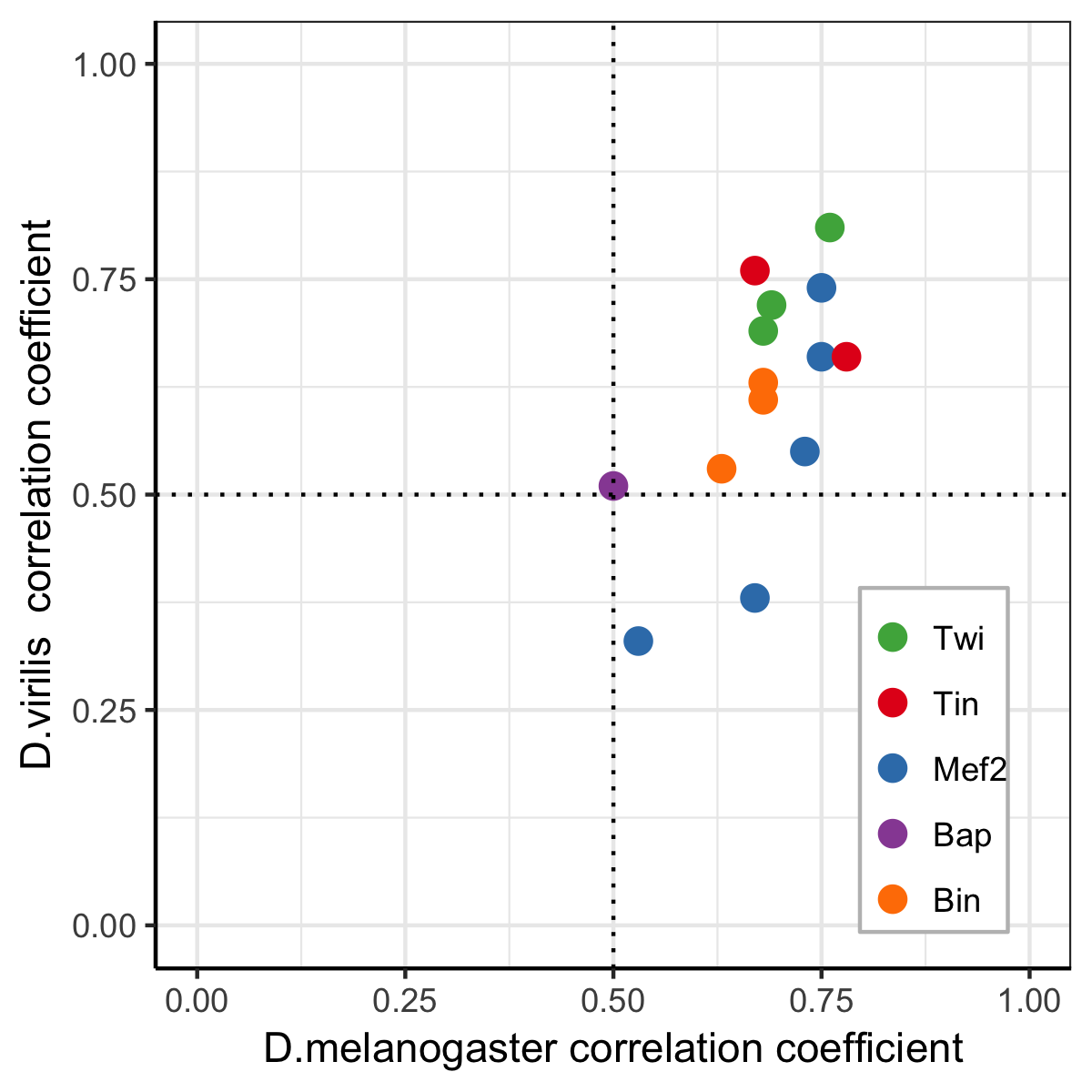


**Figure S4. STAP models accurately fit TF occupancy (ChIP) data in single species, either *D. melanogaster* (x-axis) or *D. virilis* (y-axis).** For each TF-time point condition, average Pearson correlation coefficient from 5-fold cross-validation is shown.

**Figure S5. Performance of accessibility-based preditors.** (A) Models using accessibility data from ChIP-matched stage perform nearly as well as models aggregating accessibility data from all available stages. We recognized that using data from multiple time points may inflate the strength of relationship between accessibility and binding changes, leading to unfair comparisons with motif-based predictions of ΔChIP. We therefore compared accessibility-based SVR models that use all three timepoints with models using only the time point matching the ChIP data. We found that for six of nine TF-TP conditions the two models yield equal correlations, while for three conditions the correlation is slightly better when utilizing multiple time points. (B) Performance of accessibility-based predictors is better at early time points. (Adopted from Figure 3B in main text, sorted by time points.)


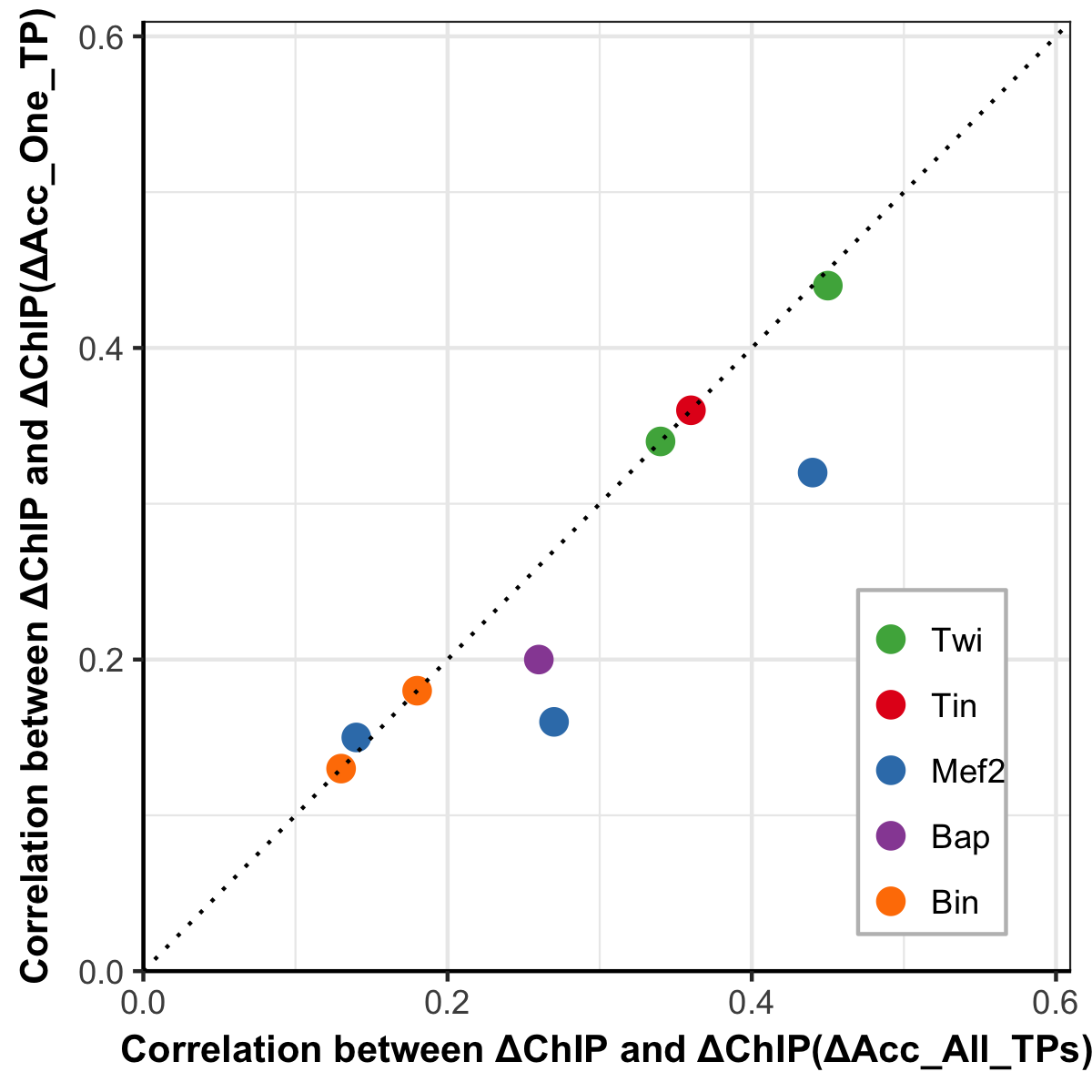

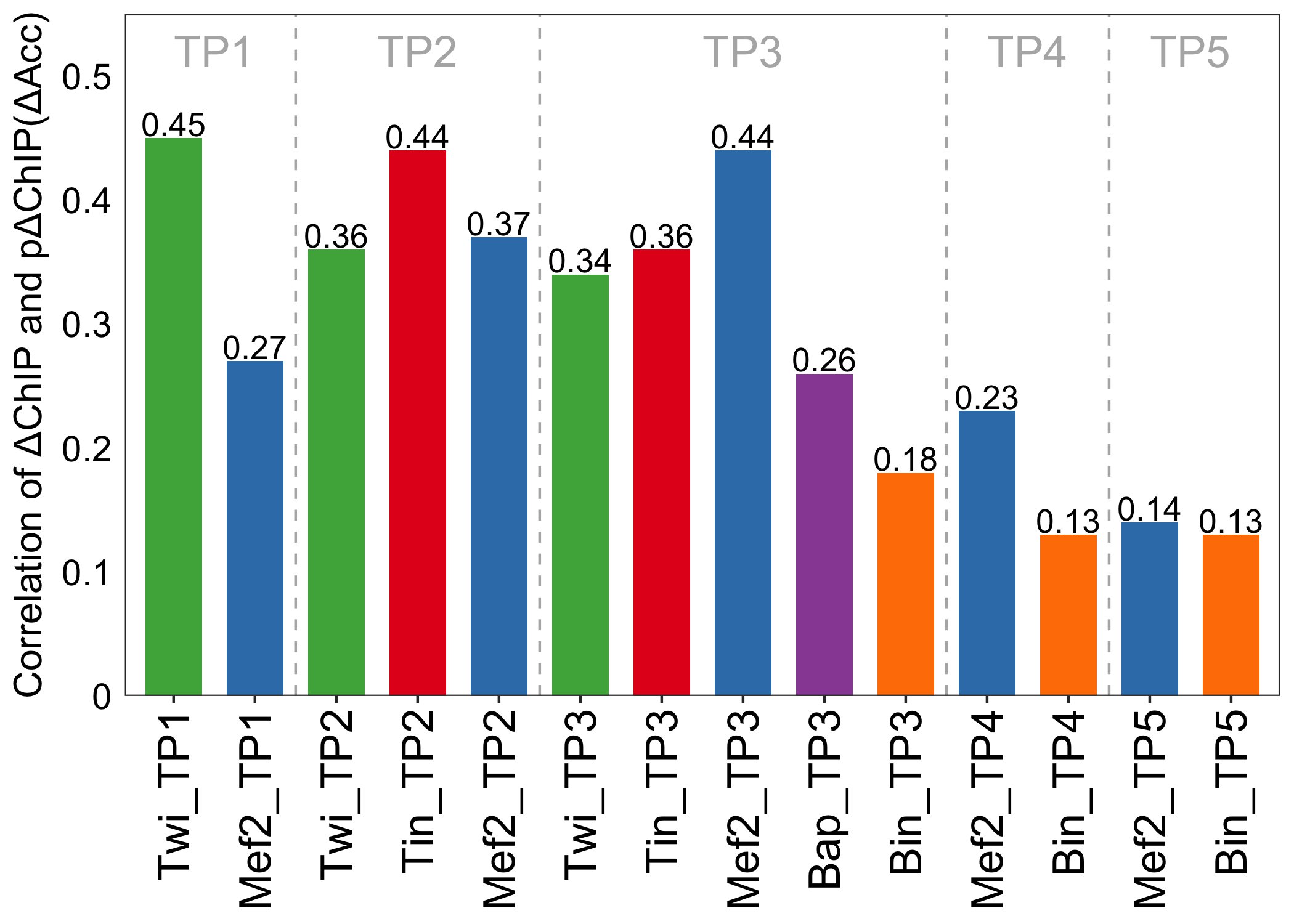


**A**

**B**


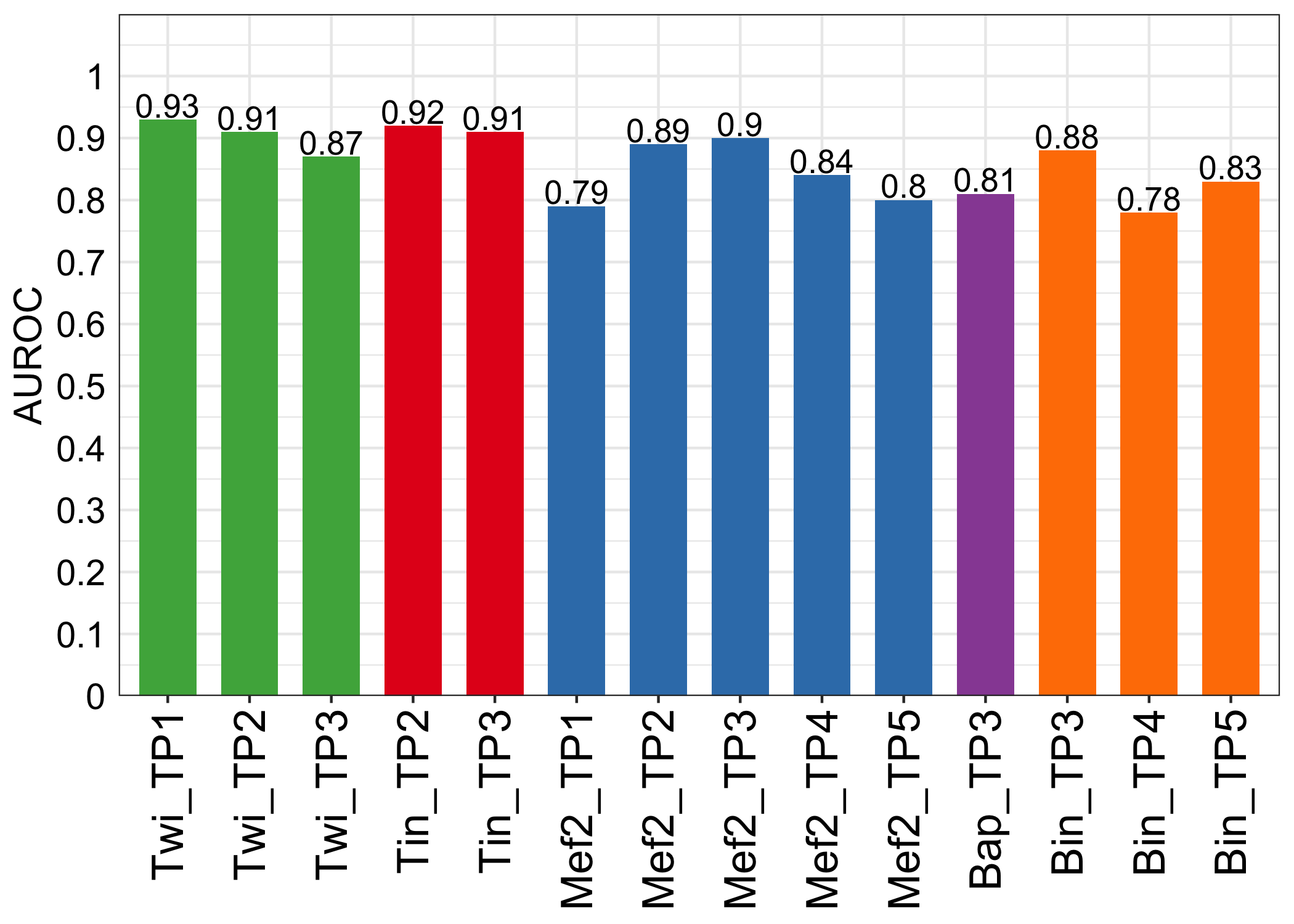


**Figure S6. AUROC measure of classification accuracy.** Predicted ΔChIP based on motif changes and accessibility changes (pΔChIP(ΔSTAP+ΔAcc)) is used to classify enhancer pairs with the greatest increase in TF binding versus those with the greatest decrease in binding.


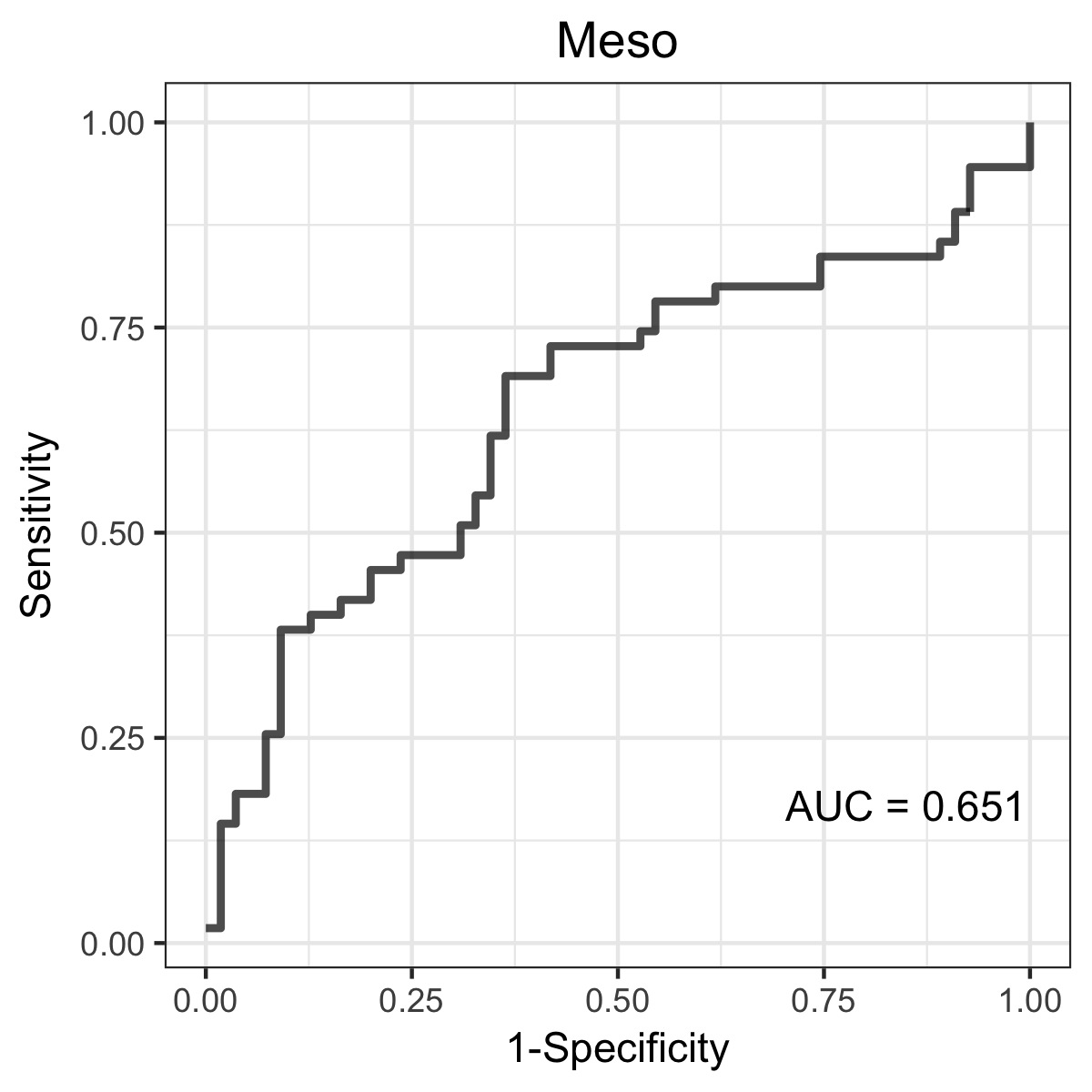

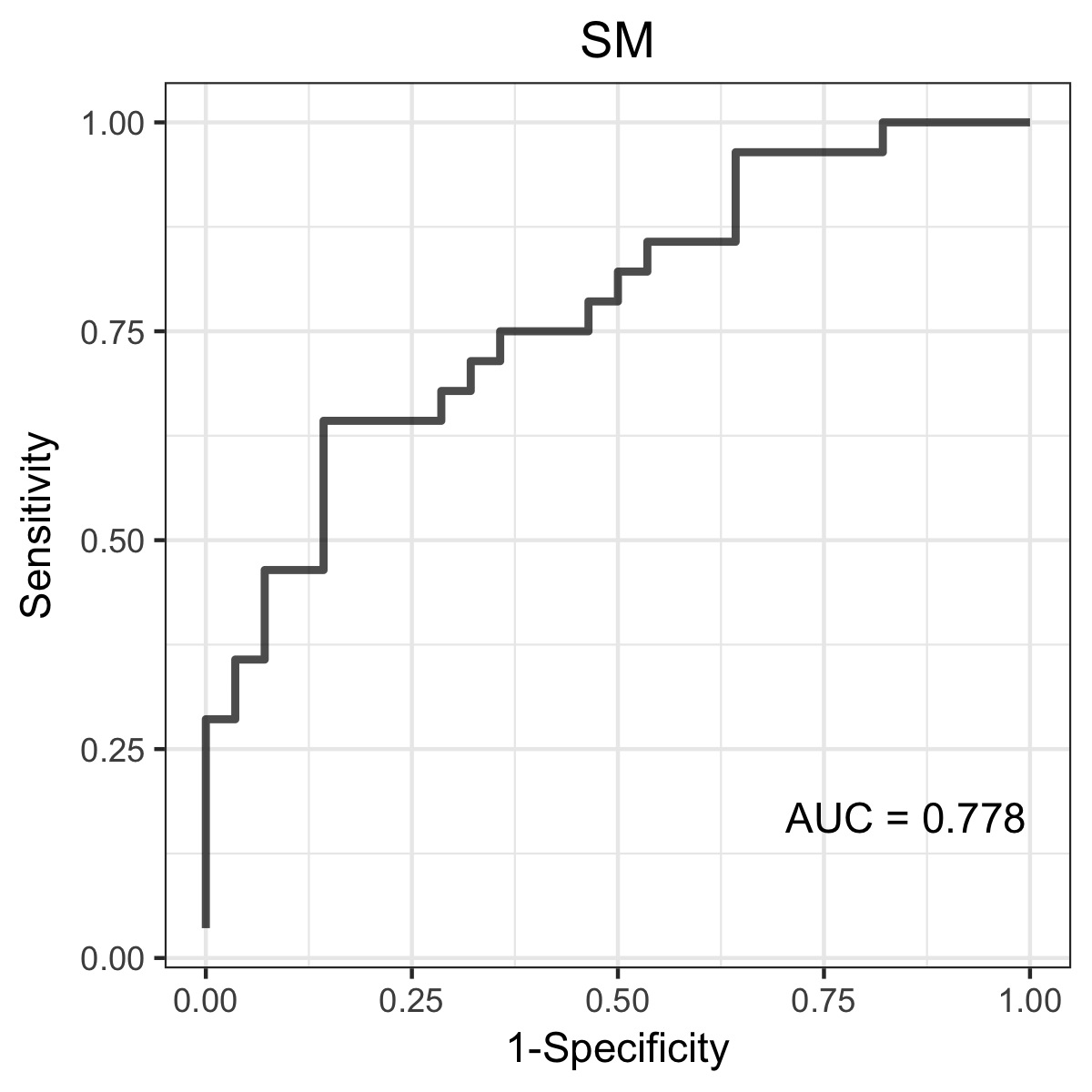

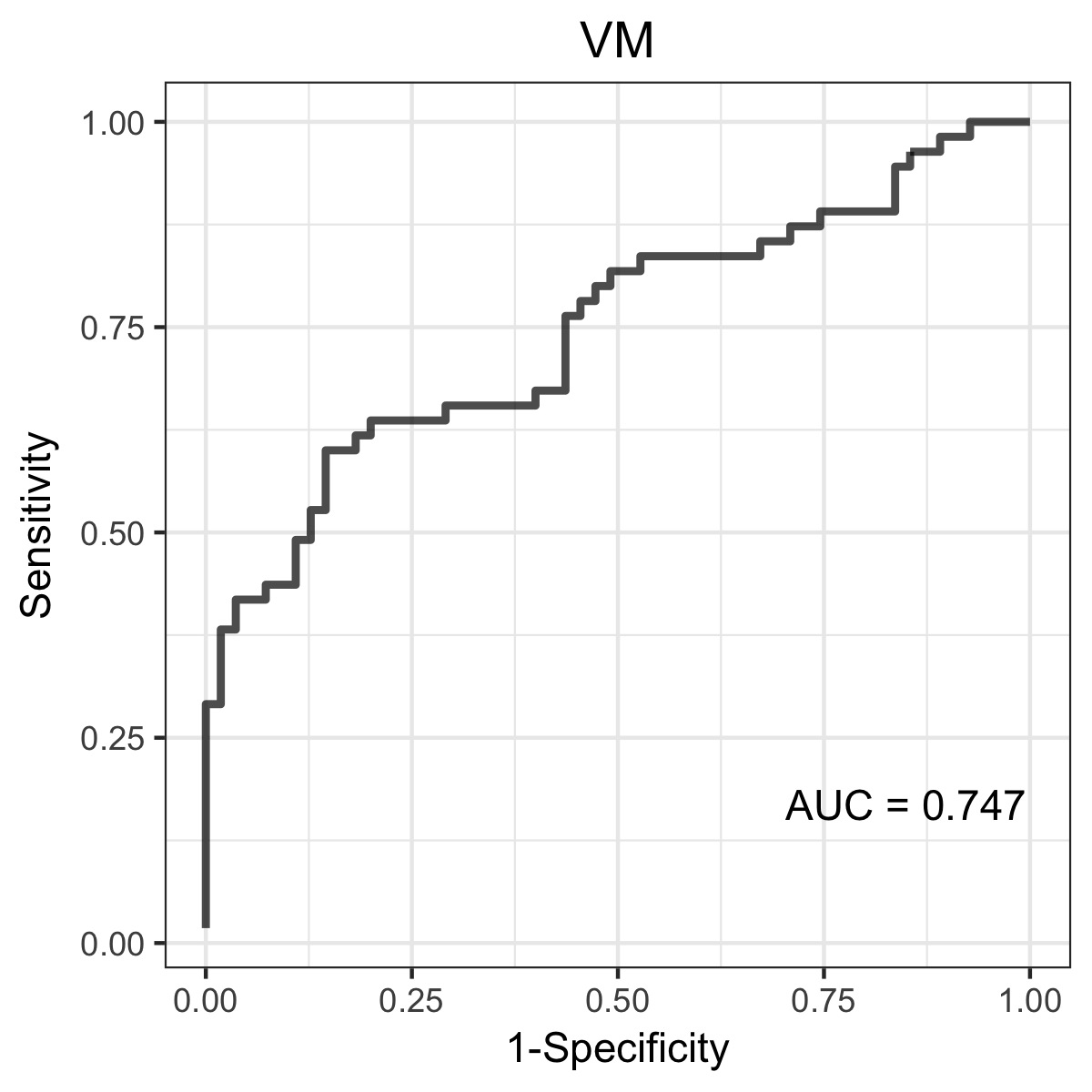


**Figure S7. ROC plots for delineating how well** $\hat{{\boldsymbol{\Delta}\boldsymbol{A}}_{\boldsymbol{C}}}$ **values can classify high versus low *ΔA_C ­_* enhancer pairs in each activity class.** $\hat{{\Delta A}_{C}}$ values are based on *D. virilis* ChIP score profiles imputed from *D. melanogaster* scores and predictions of binding change.


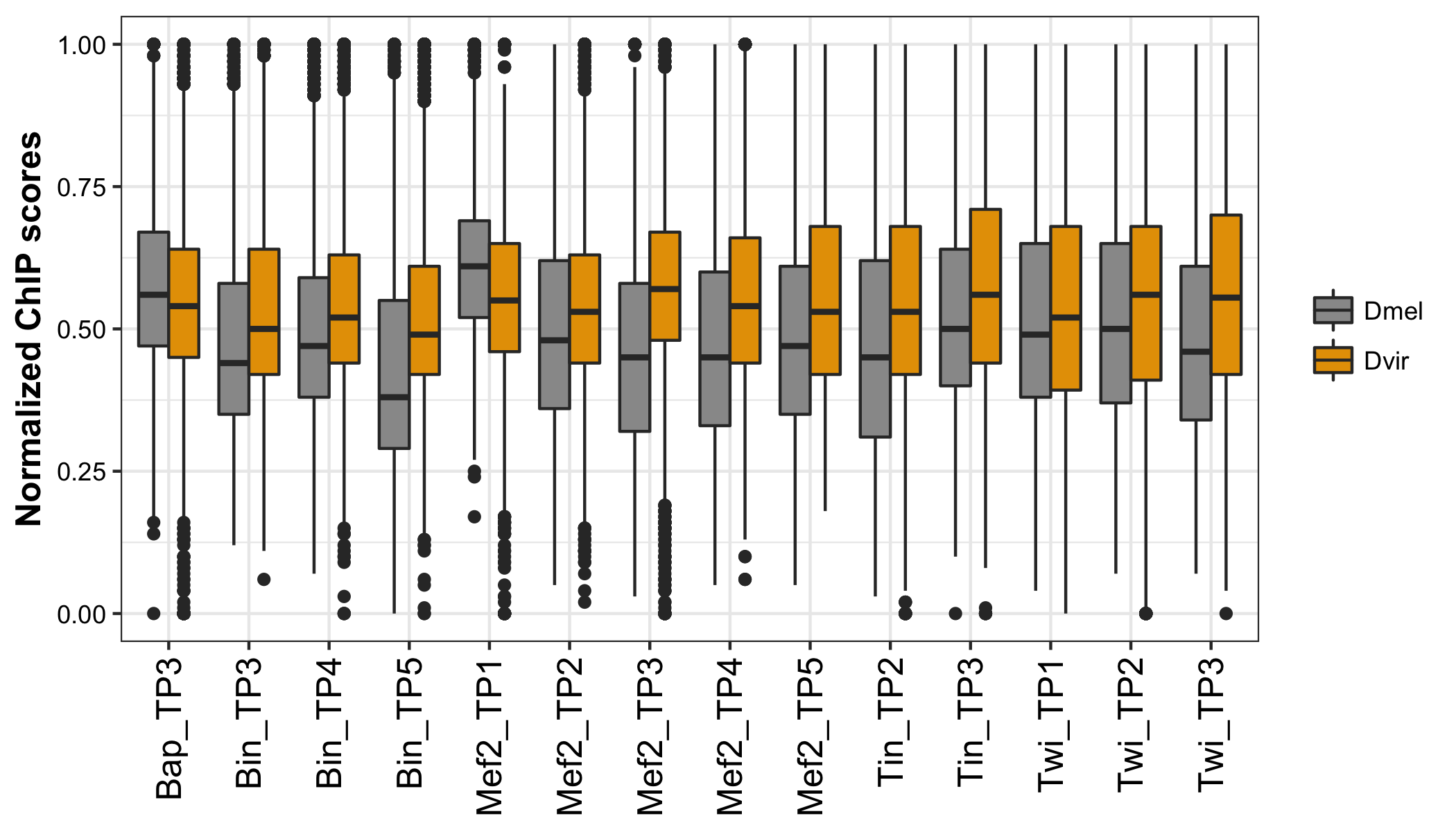


**Figure S8. Normalized ChIP scores in *D. melanogaster* and *D. virilis* show similar distributions for all 14 TF-time point conditions.**

**
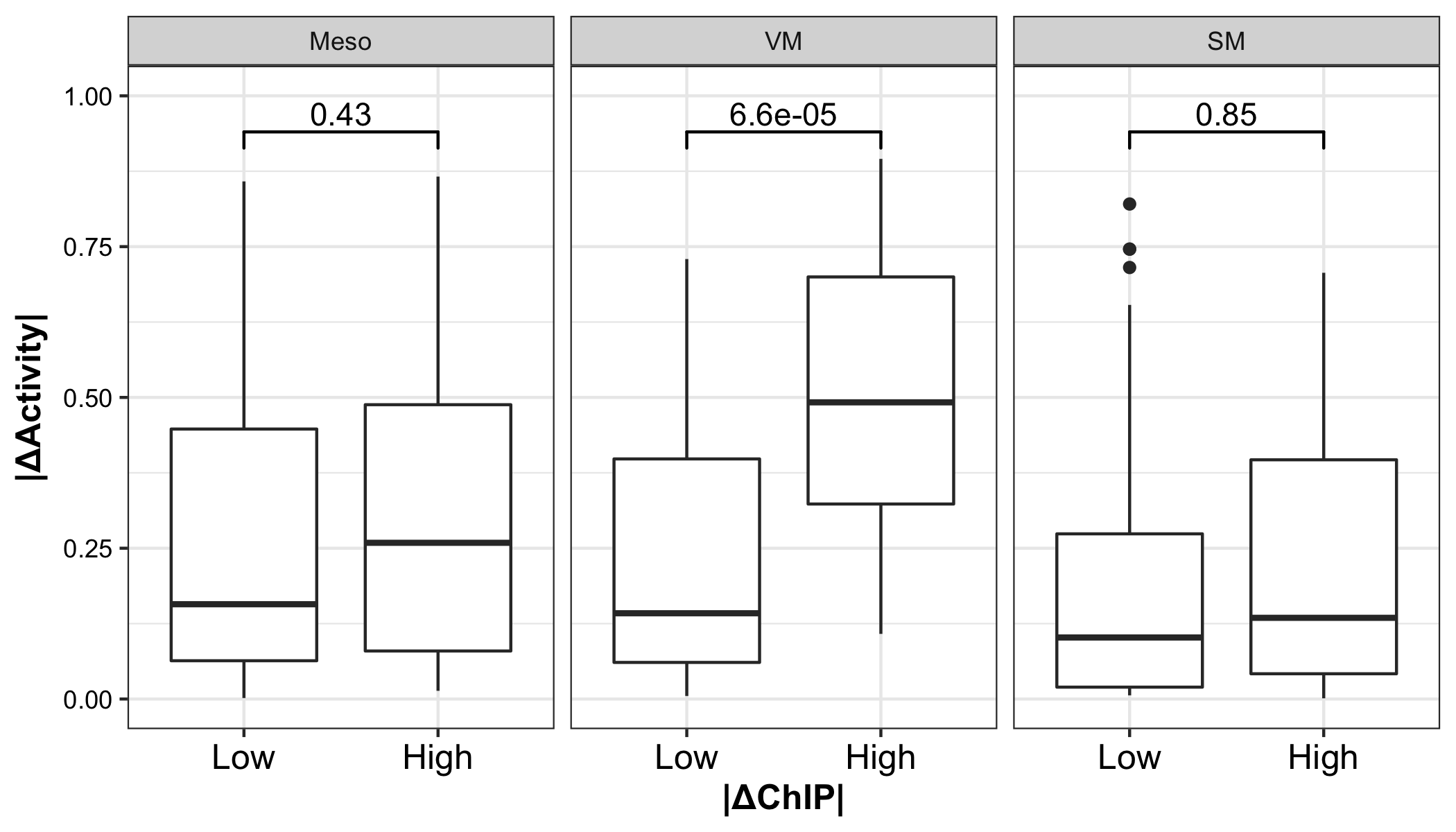
Figure S9. Change in enhancer activities.** Relationship between change of TF binding and model-based change of enhancer activity, examined through 223 experimentally characterized enhancers. For each spatiotemporal expression domain, *D. melanogaster* enhancers with experimentally validated activity in that domain are considered, along with their *D. virilis* orthologs. Enhancer pairs are divided into “High” and “Low” TF binding change, based on sum of ΔChIP scores for all TFs. Change in predicted enhancer activity (*ΔA_C_* , see text) is then compared between these two classes.

**Text S1. Relationship between total change in TF binding and predicted activity changes**

With an accurate computational model for predicting enhancer activity in hand, we examined TF binding changes between orthologous enhancers in a more contextually informed manner. We began with *D. melanogaster* enhancers that have experimentally confirmed activity in any of the three spatio-temporal expression classes (e.g. ‘C’) and calculated the regulatory activity A_C_ of each enhancer and of its *D. virilis* ortholog, based on their respective ChIP score profiles. We regarded the difference between these two A_C_ values (*ΔA_C_* = A_C_(D.mel) – A_C_(D. vir)) as an estimate of the change in regulatory activity (specific to class C) between the orthologous enhancers. We then calculated, for each orthologous enhancer pair, the sum of (absolute values of) ΔChIP scores across 14 TF:TP conditions, and used these to categorize the enhancer pairs into two groups of “High” and “Low” change in TF binding (top and bottom 25% respectively), and compared the *ΔA_C_* values between these groups (Supplementary Figure S9).

For the ‘VM’ class, we noted that the enhancer pairs with greater divergence in TF binding (‘High’ group) tend to exhibit greater change in predicted enhancer activity (P-value = 6.6E-5, Student’s t-test). On the other hand, for ‘Meso’ and ‘SM’ classes, the two groups exhibit similar distributions of *ΔA_C_*, suggesting that enhancer activities in these two expression classes are relatively robust to TF occupancy changes. This latter finding is in agreement with our previous study (Khoueiry, et al. 2017), where we assessed the impact of evolutionary changes in TF binding on enhancer activity through *in-vivo* enhancer activity assays, and found five out of seven orthologous enhancer pairs to have conserved activity despite high divergence in TF binding events. The use of activity prediction models developed in the current study allowed us to extend such assessment to 223 experimentally characterized enhancers from *D. melanogaster*, and confirm the finding that observed changes in TF binding at these enhancers may not have a functional impact.

**REFERENCES**

Khoueiry P, et al. 2017. Uncoupling evolutionary changes in DNA sequence, transcription factor occupancy and enhancer activity. eLife 6.
